# Diabetes-associated central nervous system mucormycosis with delayed diagnosis: a case report

**DOI:** 10.3389/fneur.2025.1596136

**Published:** 2025-05-06

**Authors:** Geng Jia, Changchun Yang, Yi Feng

**Affiliations:** First People's Hospital of Changzhou, Changzhou, China

**Keywords:** *Rhizopus oryzae*, CNS mucormycosis, diabetes mellitus, diabetic ketoacidosis, early diagnosis, nasal lesion

## Abstract

**Background:**

Central nervous system (CNS) mucormycosis is a rare, life-threatening fungal infection predominantly affecting diabetic and immunocompromised patients. Early diagnosis remains challenging, resulting in poor outcomes. We report a case highlighting the diagnostic challenges and rapid progression in a patient with uncontrolled diabetes and COVID-19 infection.

**Case presentation:**

A 31-year-old male with untreated type 2 diabetes mellitus presented with severe pneumonia due to COVID-19 infection. His condition rapidly deteriorated, developing CNS symptoms and characteristic nasal lesions. Cerebrospinal fluid (CSF) analysis revealed lymphocytic pleocytosis, high protein, and low glucose. Metagenomic next-generation sequencing (mNGS) of CSF confirmed *Rhizopus oryzae* infection. Despite initiating amphotericin B therapy, his condition worsened.

**Results:**

The patient succumbed to progressive multi-organ failure secondary to disseminated mucormycosis. This case emphasizes the significance of uncontrolled diabetes and COVID-19 as critical risk factors and highlights the diagnostic utility of CSF mNGS.

**Conclusion:**

Prompt recognition of risk factors, early utilization of advanced diagnostic methods, and aggressive treatment are essential to improve outcomes in CNS mucormycosis.

## 1 Introduction

Mucormycosis is a rare but often fatal invasive fungal infection primarily caused by Mucor species, with an incidence rate of 0.005 to 1.7 per million individuals (1, 2). These opportunistic fungi typically colonize the nasal mucosa, paranasal sinuses, or skin, but can cause aggressive infections in immunocompromised patients, especially those with uncontrolled diabetes, diabetic ketoacidosis (DKA), or corticosteroid use (3–5). Among the various clinical forms, rhino-orbital-cerebral and CNS mucormycosis are the most lethal, characterized by vascular invasion, thrombosis, and rapid tissue necrosis (6).

Early diagnosis is often delayed due to non-specific symptoms and the rapid disease course. The rising incidence of mucormycosis in patients with metabolic disorders underscores the importance of early recognition and management. CNS involvement is particularly devastating, with high mortality even under treatment.

Here, we report a case of CNS mucormycosis in a young male with uncontrolled diabetes, highlighting the diagnostic challenges, clinical progression, and poor outcome. This case emphasizes the need for timely intervention, high clinical suspicion in high-risk patients, and early multidisciplinary management, particularly in neurological and endocrinology settings.

## 2 Case presentation

**Figure 1:**
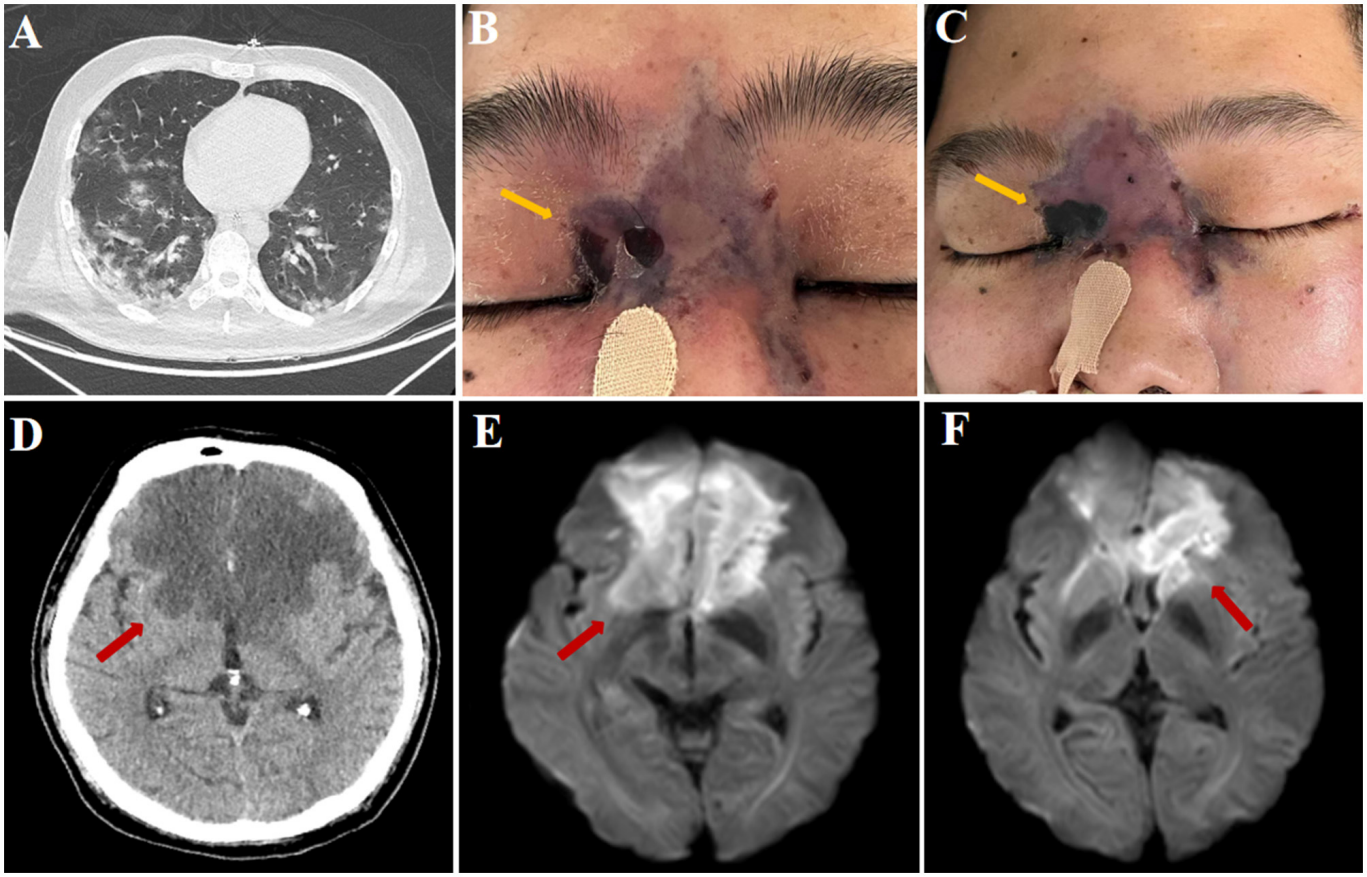
Chest computed tomography (CT) scan revealing multifocal ground-glass opacities and patchy consolidations distributed bilaterally, predominantly involving peripheral pulmonary regions **(A)**. Clinical photograph demonstrating purple-black discoloration of the nasal root with localized areas of necrotic skin forming characteristic black eschars resembling anthrax (yellow arrow), consistent with cutaneous manifestations of mucormycosis **(B)**. Clinical photograph illustrating progressive enlargement of the affected skin area at the nasal root, with a notable expansion of the characteristic black, anthrax-like necrotic eschar (yellow arrow), a hallmark of cutaneous mucormycosis progression **(C)**. Cranial computed tomography (CT) revealing bilateral hypodense lesions in the frontal lobes (red arrow), suggestive of ischemic infarction **(D)**. Diffusion-weighted imaging (DWI) of the brain demonstrating hyperintense lesions in bilateral frontal lobes (red arrow), indicative of acute ischemic infarction **(E, F)**.

We report the case of a 31-year-old male patient, unmarried, who was admitted to the hospital on January 4, 2023, with a 1-week history of cough, sputum production, and fever. He had a 7-year history of type 2 diabetes mellitus, diagnosed previously but was non-compliant with medical management, and had not received regular followup or pharmacological treatment. Upon admission, the physical examination revealed a temperature of 38.7°C, pulse rate of 112 beats per minute, respiratory rate of 25 breaths per minute, and blood pressure of 138/80 mmHg. The patient was lethargic, with a Glasgow Coma Scale (GCS) score of 13 (E3V4M6: Eye opening 3, Verbal response 4, Motor response 6). No focal neurological deficits were noted initially. His pupils were 3 mm in diameter with sluggish light reaction. Bilateral crackles were auscultated in the lungs, and limb muscle strength was graded at 4/5. Physiological reflexes were intact, and pathological signs were absent. Chest CT showed bilateral lung inflammation (Figure 1A). Laboratory tests revealed a white blood cell count of 24.2 × 10^9^/L, with 93.3% neutrophils. Arterial blood gas analysis showed a blood glucose level of 22.3 mmol/L, significant elevation of ketone bodies, and metabolic acidosis (pH 7.22, HCO3- 14.2 mmol/L). Serum sodium was 135 mmol/L, and potassium was 4.2 mmol/L.

HbA1c upon admission was 13.2%, indicating chronically poor glycemic control. The COVID-19 test returned positive. Due to severe pneumonia and confirmed COVID-19 infection, the patient was directly admitted to the COVID-19 isolation ward, and no head CT was performed at admission. After admission, the patient was empirically treated with intravenous piperacillintazobactam (4.5 g every 8 h), antiviral agents, corticosteroids, blood glucose management, fluid resuscitation, acidosis correction, and nutritional support. On January 6, the patient progressed into a coma and was transferred to the neurological intensive care unit (NSICU), presenting bilateral pupil dilation (4 mm) with absent light reflex. Neck stiffness was present, and the limbs showed flexion in response to painful stimuli. Swelling was observed at the nasal root, with the skin over the area appearing purple-black (Figure 1B). Head CT revealed bilateral frontal hypodensities (Figure 1D), and MRI confirmed the formation of acute infarcts in both frontal lobes (Figures 1E, F). Blood gas analysis showed a pH of 7.20, HCO3- 18.6 mmol/L, oxygen partial pressure of 68.6 mmHg, carbon dioxide partial pressure of 18.3 mmHg, blood glucose of 17.3 mmol/L, and lactate of 3.8 mmol/L. The patient was intubated, placed on mechanical ventilation, and received life-support therapy. On January 8, a lumbar puncture was performed. The cerebrospinal fluid (CSF) analysis showed an opening pressure of 220 mmH_2_O, leukocyte count of 80 × 106/L, protein concentration of 1.2 g/L, glucose level of 1.1 mmol/L (with a corresponding blood glucose of 12.5 mmol/L), and chloride level of 120 mmol/L. CSF was sent for metagenomic next-generation sequencing (mNGS). On January 10, mNGS confirmed *Rhizopus oryzae*, a species of Mucorales. On January 11, antifungal therapy with amphotericin B (5 mg/kg/day) was initiated. Throughout his hospitalization, the purple-black skin lesion at the nasal root progressively expanded, leading to local skin breakdown and black eschar formation (Figure 1C). Due to the patient's rapidly deteriorating condition, biopsy or tissue swab of the lesion was not performed. Sputum culture identified Enterobacter cloacae, and blood cultures yielded Candida parapsilosis; Mucorales were not detected in peripheral blood cultures. Despite intensive life-support therapy including mechanical ventilation, the patient' s condition continued to worsen, resulting in multiple organ failure. On January 14, given the extremely poor prognosis, his family elected to withdraw active treatment and requested discharge against medical advice. The patient passed away at home on January 15, 2023, presumably from progressive multiple organ failure secondary to disseminated fungal infection.

## 3 Discussion

Central nervous system (CNS) mucormycosis is a severe brain infection caused by *Mucor* species. CNS mucormycosis most commonly arises from local invasion of adjacent structures such as the paranasal sinuses or orbit through direct extension across the cranial base. Hematogenous dissemination is typically associated with primary infections originating in the lungs or gastrointestinal tract. The disease progresses rapidly, with a mortality rate as high as 90% (7). A key feature of Mucor infection is its ability to invade blood vessels quickly, damaging the vessel walls and causing thrombosis, which leads to local ischemia, brain infarction, hemorrhagic necrosis, and brain abscesses (5, 8). Common symptoms of CNS mucormycosis include headache, vomiting, seizures, and altered consciousness, often accompanied by severe neurological deficits. Current antifungal therapies typically have delayed efficacy, and patients often succumb due to multiple organ failure or cerebral death.

The strong link between mucormycosis and metabolic disorders, particularly diabetes mellitus and diabetic ketoacidosis (DKA) (9–11). Patients with uncontrolled diabetes (particularly those with an HbA1c level exceeding 10%), diabetic ketoacidosis, hematologic malignancies, solid organ transplantation, or prolonged corticosteroid therapy are at the highest risk for developing mucormycosis (9, 12, 13). Chronic hyperglycemia in diabetic patients impairs immune function, particularly during ketoacidosis or extreme hyperglycemia, which decreases white blood cell function and creates favorable conditions for mucormycosis. Studies show that diabetic patients, particularly those with hyperglycemia or ketoacidosis, are at greater risk for *Mucor* infections, with a higher mortality rate (3, 5). Long-term corticosteroid use is another significant risk factor, as it suppresses immune function and increases blood glucose levels, reducing the body's defense against fungi and allowing opportunistic pathogens like Mucor to cause infection (14, 15).

In this case, the patient initially presented with respiratory symptoms and subsequently developed purple-black discoloration of the nasal skin during hospitalization, an early hallmark of mucormycosis. Particularly in diabetic or immunocompromised patients, this finding is diagnostically significant but often misdiagnosed as soft tissue infection, delaying the recognition of a fungal etiology. As the disease progressed rapidly, the affected area expanded, forming black eschar with anthrax-like changes, indicative of deep tissue necrosis. Nasal lesions serve as critical diagnostic and prognostic markers for mucormycosis (16). Failure to promptly identify and address these lesions can result in rapid infection spread to the orbit, facial tissues, and central nervous system, significantly increasing mortality (17). Nasal skin lesions, while highly characteristic of mucormycosis, may also present in other invasive fungal infections such as aspergillosis and candidiasis, especially among immunocompromised patients. Therefore, heightened clinical vigilance and timely microbiological assessment are essential for accurately distinguishing mucormycosis from other fungal infections exhibiting similar dermatologic features.

Early diagnosis and multidisciplinary management, including prompt antifungal treatment and surgical debridement, are critical for improving survival. Imaging studies such as MRI or CT can help assess the extent of lesions, while early fungal testing is key to confirming the diagnosis.Lumbar puncture plays a critical role in diagnosing suspected central nervous system mucormycosis, particularly when neuroimaging findings are inconclusive. Typical cerebrospinal fluid (CSF) abnormalities include elevated opening pressure, lymphocytic pleocytosis, increased protein concentration, and reduced glucose levels. However, conventional microbiological cultures frequently yield negative results. Advanced diagnostic methods, such as metagenomic nextgeneration sequencing (mNGS), markedly improve diagnostic accuracy by enabling rapid and precise identification of Mucorales species, particularly in cases where standard culture methods fail or are delayed.

Amphotericin B is the standard treatment for mucormycosis, but its slow action and significant side effects are notable drawbacks (18–20). Newer antifungal agents, such as voriconazole and posaconazole, have been used in mucormycosis treatment, though issues of resistance and variable efficacy remain (21). Surgical intervention is essential for controlling local infections and removing necrotic tissue, especially in early-stage sinus or orbital lesions, which helps prevent the infection from spreading (19).

## 4 Conclusion

CNS mucormycosis is a rare but life-threatening opportunistic fungal infection, particularly in patients with uncontrolled diabetes or immunosuppression. Early identification remains challenging due to non-specific clinical manifestations and limited diagnostic yield from conventional tests. Metagenomic next-generation sequencing (mNGS) of cerebrospinal fluid offers a valuable tool for timely and accurate pathogen detection when traditional methods fail. Moreover, prompt antifungal therapy combined with multidisciplinary collaboration is essential to optimize patient outcomes. Heightened clinical awareness and the integration of advanced molecular diagnostics are key to improving the prognosis of this devastating condition.

## Data Availability

The datasets presented in this article are not readily available because of ethical and privacy restrictions. Requests to access the datasets should be directed to the corresponding author.
